# Failure of a patient-derived xenograft for brain tumor model prepared by implantation of tissue fragments

**DOI:** 10.1186/s12935-016-0319-0

**Published:** 2016-06-10

**Authors:** Kyung-Min Kim, Jin-Kyoung Shim, Jong Hee Chang, Ji-Hyun Lee, Se-Hoon Kim, Junjeong Choi, Junseong Park, Eui-Hyun Kim, Sun Ho Kim, Yong-Min Huh, Su-Jae Lee, Jae-Ho Cheong, Seok-Gu Kang

**Affiliations:** Department of Medical Science, Yonsei University College of Medicine, 50-1 Yonsei-ro, Seodaemun-gu, Seoul, 03722 Republic of Korea; Department of Neurosurgery, Severance Hospital, Brain Tumor Center, Yonsei University College of Medicine, 50-1 Yonsei-ro, Seodaemun-gu, Seoul, 03722 Republic of Korea; Department of Pathology, Severance Hospital, Yonsei University College of Medicine, 50-1 Yonsei-ro, Seodaemun-gu, Seoul, 03722 Republic of Korea; Department of Pharmacy, Yonsei University College of Pharmacy, 85 Songdogwahak-ro, Yeonsu-gu, Incheon, 21983 Republic of Korea; Department of Radiology, Severance Hospital, Yonsei University College of Medicine, 50-1 Yonsei-ro, Seodaemun-gu, Seoul, 03722 Republic of Korea; Department of Life Science, Research Institute for Natural Sciences, Hanyang University, 222, Wangsimni-ro, Seongdong-gu, Seoul, 04763 Republic of Korea; Department of Surgery, Severance Hospital, Yonsei University College of Medicine, 50-1 Yonsei-ro, Seodaemun-gu, Seoul, 03722 Republic of Korea

**Keywords:** Glioblastoma, Model failure, Patient-derived xenograft, Primitive neuro-ectodermal tumor, Tissue fragment

## Abstract

**Background:**

With the continuing development of new anti-cancer drugs comes a need for preclinical experimental models capable of predicting the clinical activity of these novel agents in cancer patients. However existing models have a limited ability to recapitulate the clinical characteristics and associated drug sensitivity of tumors. Among the more promising approaches for improving preclinical models is direct implantation of patient-derived tumor tissue into immunocompromised mice, such as athymic nude or non-obese diabetic/severe combined immunodeficient (NOD/SCID) mice. In the current study, we attempted to develop patient-derived xenograft (PDX) models using tissue fragments from surgical samples of brain tumors.

**Methods:**

In this approach, tiny tissue fragments of tumors were biopsied from eight brain tumor patients—seven glioblastoma patients and one primitive neuroectodermal tumor patient. Two administration methods—a cut-down syringe and a pipette—were used to implant tissue fragments from each patient into the brains of athymic nude mice.

**Results:**

In contrast to previous reports, and contrary to our expectations, we found that none of these fragments from brain tumor biopsies resulted in the successful establishment of xenograft tumors.

**Conclusions:**

These results suggest that fragments of surgical specimens from brain tumor patients are unsuitable for implementation of brain tumor PDX models, and instead recommend other in vivo testing platforms for brain tumors, such as cell-based brain tumor models.

**Electronic supplementary material:**

The online version of this article (doi:10.1186/s12935-016-0319-0) contains supplementary material, which is available to authorized users.

## Background

The multiple genetic lesions and complex signaling cascades associated with the etiology and progression of cancer contribute to difficulties in understanding the biology of this disease [1]. Of the various kinds of cancers, brain tumors, especially glioblastoma (GBM), are among the most lethal [2]. Thus not surprisingly, considerable research effort has been devoted to increasing the efficacy of new treatments for GBM [3–6]. Although these efforts have been ongoing for decades, preclinical tumor models suitable for evaluating the effects of anti-cancer therapies are lacking [7–9].

A number of animal models of primary brain tumors have been developed to understand brain tumors, including chemically induced models, genetically engineered mouse models, and tumor xenograft models [8]. Among these animal models, tumor xenografts derived from human GBM cell lines have traditionally been used as a preclinical tool to understand cancer biology and therapeutic efficacy. However, GBM cell line xenografts have a number of shortcomings for preclinical use, including (i) the failure of tumor xenografts to mirror patient responses [10, 11] because they do not accurately reflect the clinical characteristics of the patient tumor, (ii) differences in pharmacokinetics between animal models and as humans [12], and (iii) the loss of biological properties of cancer cell lines during the process of establishing them [13, 14].

To overcome the shortcomings of previous tumor models and to preserve the oncological heterogeneity observed in patients, researchers are continuing to focus on new animal testing platforms for cancer. Several recent studies have reported innovative preclinical animal models generated by transplanting sectioned patient-derived tumor tissue fragments, rather than cell lines, into immunocompromised mice, such as athymic nude or non-obese diabetic/severe combined immunodeficient (NOD/SCID) mice [7, 15–18], a concept generally referred to in the literature as patient-derived xenograft (PDX) [15, 18]. Maintaining the histological characteristics and primary architecture of tumors is one of the major advantages of the PDX model [18, 19]. In addition, the propagation of tumors in successive generations of mouse hosts enables PDX cells to avoid the stressful conditions that possibly occur during cell culture [20]. These advantages make PDXs biologically stable and enable them to maintain the molecular characteristics of the primary tumors from which they were derived [20, 21]. Because of these strengths, PDXs are useful not only for the preclinical testing of drugs, but also to verify molecular changes and signaling pathways in oncology [1].

A recent series of studies in the brain tumor field has made meaningful progress in animal testing platforms using patient-derived tumorspheres [10, 22–24] and enzymatically dissociated cells [25]. However, because these previous studies used tumor cells, the resulting animal models may not satisfy the classic definition of a PDX [1, 15, 16, 18]. Making PDXs more similar to the originating GBM patient tumors may instead require injection of patient-derived tumor tissue fragments. In this context, one report concluded that PDXs formed by implanting GBM tissue fragments were similar to the original tumors of GBM patients [26]. In the present study, we attempted to develop PDX models using tissue fragments from surgical samples of brain tumors using two different direct injection methods: a cut-down syringe and a pipette.

## Methods

### Patient population

Eight patients with primary brain tumors, including seven glioblastoma (GBM) patients and one primitive neuroectodermal tumor (PNET) patient, treated at our institution between June 2013 and October 2013, were included in this study (Table 1). All patients were histologically diagnosed and graded by neuro-pathologists according to 2007 World Health Organization (WHO) classification criteria [27]. O-6-methylguanine-DNA methyltransferase (MGMT) promotor methylation and isocitrate dehydrogenase (IDH)-1 mutations were assessed by polymerase chain reaction (PCR) and immunohistochemistry (IHC). Epidermal growth factor receptor (EGFR) amplification and loss of heterozygosity (LOH) at chromosomes 1p and 19q were evaluated by fluorescent in situ hybridization (FISH). P53 and ki-67 were examined by IHC. All patients provided written informed consent, and the study was approved by the Institutional Review Boards of our institution.

**Table 1:** Patient characteristics

	Age	Sex	WHO grade	Pathology	MGMT^a^	IDH1^b^	P53	1p/19q	Ki-67 L.I.	EGFR^g^	No. of implanted mice
Case #1	24	M	IV	GBM^c^	Methylation	Wild type	Wild type	Intact	5 %	0	3
Case #2	61	M	IV	GBM^c^	Unmethylation	Wild type	5 %	LOH/intact	Focal 20–30 % and overall 5 %	3+	3
Case #3	24	M	IV	GBM^c^	Unmethylation	Wild type	80–90 %	Intact	50 %	2–3+	3
Case #4	50	M	IV	GBM^c^	Methylation	Mutation	NA	NA	NA	NA	3
Case #5	62	F	IV	GBM^c^	Methylation	Wild type	Wild type	Intact	5–6 %	3+	4
Case #6	11	M	IV	GBM^c^	Unmethylation	Wild type	50–60 %	Intact	70–80 %	0	3
Case #7	26	M	IV	GBM^c^	Unmethylation	Wild type	50–60 %	LOH^f^	40–50 %	2–3+	3
Case #8	1.3	M	IV	PNET^d^	NA^e^	NA	NA	NA	50–60 %	NA	3

^a^ O(6)-methylguanine methyltransferase

^b^ Isocitrate dehydrogenase 1

^c^ Glioblastoma

^d^ Primitive neuroectodermal tumor

^e^ Non-available

^f^ Loss of heterozygosity

^g^ Epidermal growth factor receptor amplification

### From fresh tumor specimen to small tissue fragments

Specimens from GBM patients were freshly obtained from the operating room, placed in sterile centrifuge tubes (SPL Life Sciences Co., Ltd, Seoul, Korea) in ice, and then chopped with a sterile stainless steel surgical blade (Paragon, Sheffield, UK) and suspended in phosphate-buffered saline (PBS; Mediatech, Manassas, VA, USA) within 30 min of being sent from the surgical room. Fragmented specimens, approximately 3 mm^3^ in size (Aliquots of 3 μl of suspended tumor fragments were prepared for injection), were prepared for each sample [15, 26].

### Animals

Male athymic nude mice (Central Lab Animal Inc., Seoul, Korea), aged 4–8 weeks, were used in this study. Mice were housed in micro-isolator cages under sterile conditions and were observed for at least 1 week before study initiation to ensure proper health. Lighting, temperature, and humidity were centrally controlled. All experimental procedures were approved by the Institutional Animal Care and Use Committee of Yonsei University College of Medicine.

### Orthotopic xenograft of patient-derived brain tumor tissue fragments

**Fig. 1:**
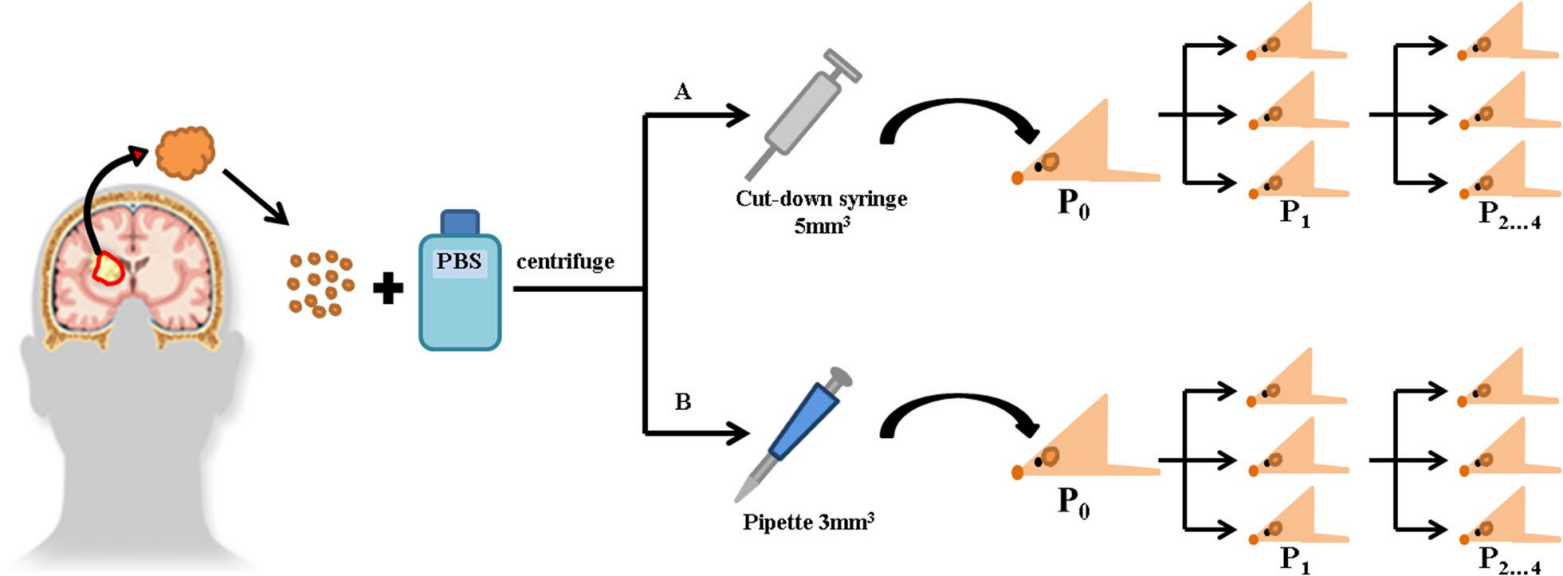
Methods for developing PDX mouse models. Athymic mice received patient-derived surgical brain tumor tissue fragments (~3 mm^3^) via two different injection methods: 21 gauge cut-down syringe and pipette

Brain tumor tissue fragments from each patient (n = 8) were directly injected into mice (n = 3/group) using two methods: a 21 gauge, cut-down syringe (Korea Vaccine Co., Ltd., Gyeonggi-do, Korea), and a pipette (Gilson, Middleton, WI, USA) (Fig. 1). Mice were first anesthetized with a solution of Zoletil (30 mg/kg; Virbac Korea, Seoul, Korea) and xylazine (10 mg/kg; Bayer Korea, Seoul, Korea), delivered intraperitoneally. Then, a hole was drilled in the right side of the skull at the implantation position (2.5 mm lateral from sagittal suture, 1 mm anterior from coronal suture) using a small, hand-controlled twist drill (Plastics One, Roanoke, VA, USA). Thereafter, patient-derived brain tumor tissue fragments (~3 mm^3^) were implanted to a depth of 4.5 mm in the right frontal lobe of nude mice via an inserted cut-down syringe or pipette.

## Results

**Fig. 2:**
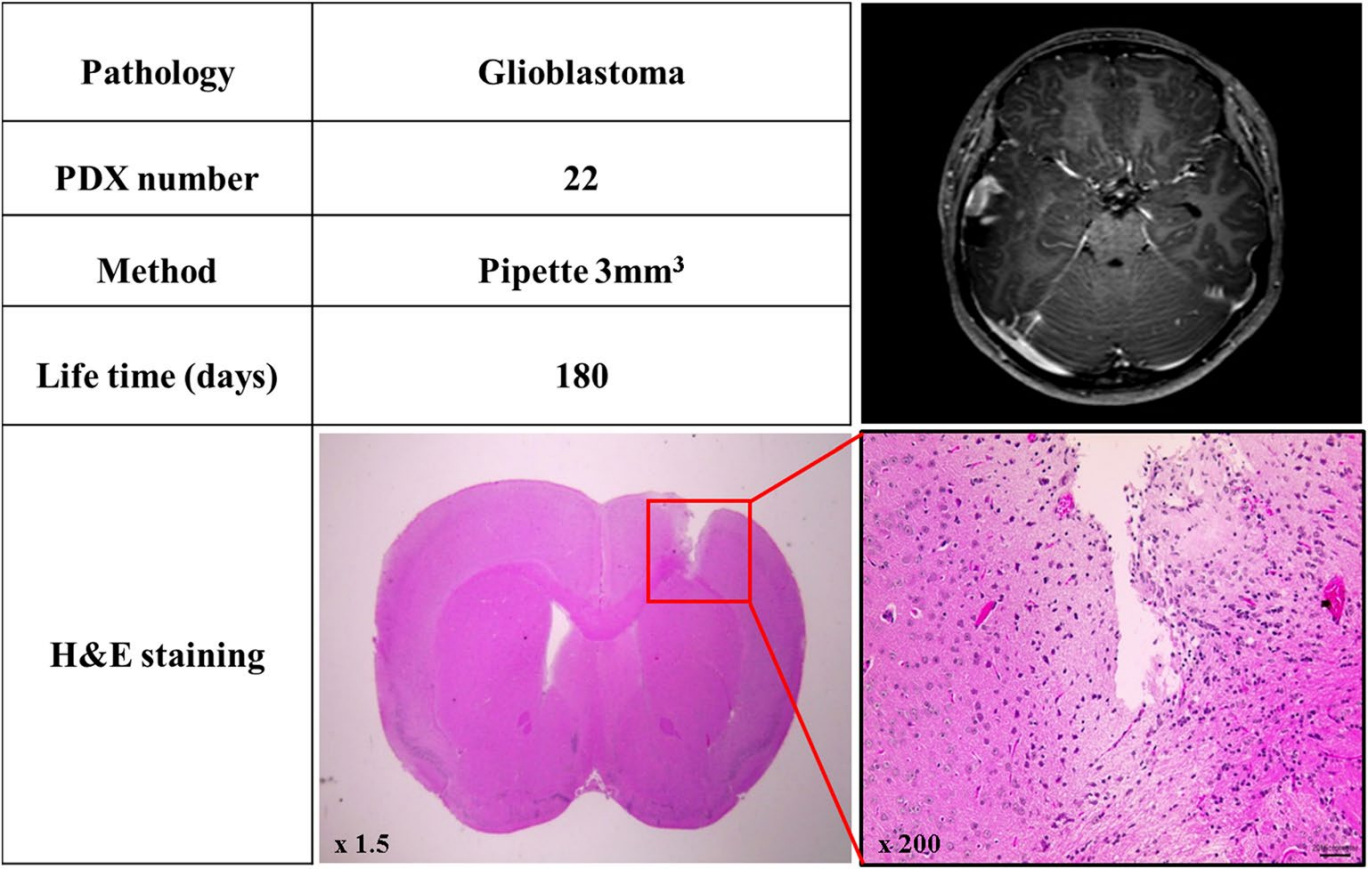
Example of a PDX data set. Each data set included the pathology of the donor patient, an MRI (T1 axial enhancement) image of the donor patient, PDX number, injection method, lifetime of the PDX, and an H&E-stained histological section of PDX brain tissue. This data set corresponds to that for tumor tissue fragments from a glioblastoma patient with a T1 contrast-enhanced lesion in the *right* temporoparietal lobe implanted into PDX mouse #22 through a pipette. No xenograft patient-derived tumor tissue fragments were detected in H&E-stained histological sections of brain tissue from this mouse, as confirmed by Neuro-pathologists

Patient-derived xenograft (PDX) models were prepared for eight brain tumor patients (Table 1) by implanting tiny tissue fragments biopsied from each patient into the brains of athymic mice (n = 3/group) using the two injection methods—cut-down syringe and pipette—described in “Methods” section. A dataset was obtained for each PDX that included a pathological assessment and magnetic resonance imaging (MRI; T1 axial enhancement) of the corresponding donor, the injection method, the lifetime of the resulting PDX, and images of hematoxylin and eosin (H&E)-stained histological sections of PDX brain tissue (Fig. 2).

The neuro-pathologists examined each PDX brain. Tissues were fixed in 4 % phosphate-buffered paraformaldehyde for 24 h, embedded in paraffin, sectioned, and stained with H&E to examine whether the xenografted tumor tissue fragments had been successfully established. A total of 17 PDXs were prepared by injecting tissue fragments via a cut-down syringe and 8 PDXs were prepared by injecting with a pipette. Among these PDXs, four in the cut-down syringe group and one in the pipette group died because of surgical complications. Apart from these dead PDXs, no PDX-bearing patient-derived brain tumor was detected, yielding an overall establishment rate of 0 %. The neuro-pathologists verified that the PDX brain tissue sections showed inflammation and was thus unlikely to be necrotic (Table 2).

**Table 2:** Tissue fragment implantation results

Method	No. of mice	No. of deaths^a^	No. of non-tumor–bearing mice	Establishment rate (%)
Cut-down syringe	17	4	13	0
Pipette	8	1	7	0

^a^ Surgical complications

## Discussion

Previous studies have reported testing platforms that attempt to recapitulate the properties of patients’ tumors using tumorspheres [10] or dissociated cells [25]. One additional previous study also suggested that a xenograft formed from glioblastoma (GBM) fragments inserted into the brain was similar to that of the originating GBM tumor [26]. On the basis of these results, we hypothesized that brain tumor patient-derived xenograft (PDX) models could be successfully established through direct implantation of tumor tissue fragments. To create these models, we inserted tiny tumor tissue fragments (~3 mm^3^), biopsied from brain tumor patients, into the brains of athymic mice using two injection methods: a cut-down syringe and a pipette. Among these PDXs, ~3 % died due to surgical complication. Inflammation was the only fatal surgical complication observed, and mice subject to it died an average of 5 days post-op. The post-op survival of all other mice was longer than 6 months. An analysis of each PDX brain tissue section to determine whether the xenografted tumor tissue fragments successfully became established unexpectedly revealed a tumor-establishment rate for xenografted brain tumor tissue fragments of 0 %. Thus, contrary to our hypothesis, we could not develop brain tumor PDXs from tumor tissue fragments, considered an ideal method for immortalization of a patient’s cancer.

Considerable effort has been devoted to identifying potential animal hosts for xenotransplantation of human cells for use in the development of animal models for human diseases. As a consequence, a number of immunocompromised mouse models, including athymic nude mice and severe combined immunodeficient (SCID) mice, have been developed. Motivated by previous research [26], we used T cell deficient athymic nude mice as xenografts animal subjects. However, these mice still have functional B cells, which could cause failure of engraftment. Accordingly, a change in animal model to SCID mice, which are defective in functional T and B cell, could facilitate engraftment. One report has also suggested NOD/SCID/γ_c_^null^ mice, a modified SCID mouse with multiple immunological dysfunctions, as an animal recipient for improving the engraftment success of xenografts [28].

From the standpoint of transplanting tumors most like the original, our study is similar to a previous report [26]. Fei et al. injected 2 mm^3^ of tumor tissue fragments per mouse in a total maximum volume of 20 μl injected cell suspension [26, 29, 30]. Here, we implanted 3 mm^3^ of suspended tumor tissue fragments per mouse, in an effort to increase the amount of tissue fragments implanted. In both studies, the tumor tissue fragments were injected into mice within 5 min (from anesthesia to closure of skull hole). In our study, it took about 15 min to transfer the samples from the operating room to the laboratory, and 15 min to chop the samples, but no specific sample movement time was reported in the previous study. We used a cut-down syringe and pipette to insert tumor tissue fragments, whereas the previous study used a trocar for injection. This single known difference in methodology may have caused differences in the total volume of injected tissue fragments, resulting in the implantation of a relatively small number tumor cells in our study. It might also have produced differences in injection pressure or speed that ultimately proved detrimental.

In this study, no PDX-bearing patient-derived brain tumor was detected. It is possible that this might reflect a loss of cell viability, perhaps due to our brief placement of fresh patient-derived brain tumor tissue fragments in ice, or the suspension of brain tumor tissue fragments in PBS (which was required because otherwise the chopped tissue fragments were too sticky). We do not believe, however, that there was any issue with loss of cell viability. We used PBS instead of medium to exclude any effects from factors found in the medium. Specimens were chopped with a surgical blade and suspended in PBS within 30 min being sent from the surgical room, and were then injected within 30 min of the chopping step. Moreover, we performed cell culture using specimens placed in ice and suspended with PBS, as described in our protocol, and successfully obtained cultured tumor cells (Additional file 1: Figure S1). Additional method for cell isolation was described in Additional file 2. Finally, previous reports have described the successful isolation of viable cells using the same method [23, 24]. Thus, we believe that our patient-derived brain tumor tissue fragments maintained their viability during the processing steps.

The 0 % tumor-establishment rate for xenografted brain tumor tissue fragments may be attributable to these differences. However, despite the differences between our method and the trocar injection system, the basic concept of both methods is similar. Thus, notwithstanding a previous report of GBM PDX models prepared using athymic mice, our results suggest that tissue fragments of surgical brain tumor specimens from patients may be unsuitable for implementation of brain tumor PDX models. Previous reports described the successful development of brain tumor xenograft models using the same methods applied in the present work [23, 24, 31]. Though the methods in this study were proved through previous reports, alteration of some method conditions such as alternative tissue preparation, injection techniques, and selection of immunodeficient animals can prove more successful in establishing brain tumor PDX models.

## Additional files

10.1186/s12935-016-0319-0 Culture of tumor spheres from processed specimens.
10.1186/s12935-016-0319-0 Method for culture of tumor spheres from processed specimens.

## Abbreviations

PDX: patient-derived xenograft
GBM: glioblastoma
NOD-SCID: non-obese diabetic/severe combined immunodeficient
PNET: primitive neuroectodermal tumor
