# Supplementary figures and images for: Failure of a patient-derived xenograft for brain tumor model prepared by implantation of tissue fragments
Source: Cancer Cell Int. 2016 Jun 10;16:43. doi: 10.1186/s12935-016-0319-0 (PMC4901492; doi:10.1186/s12935-016-0319-0)

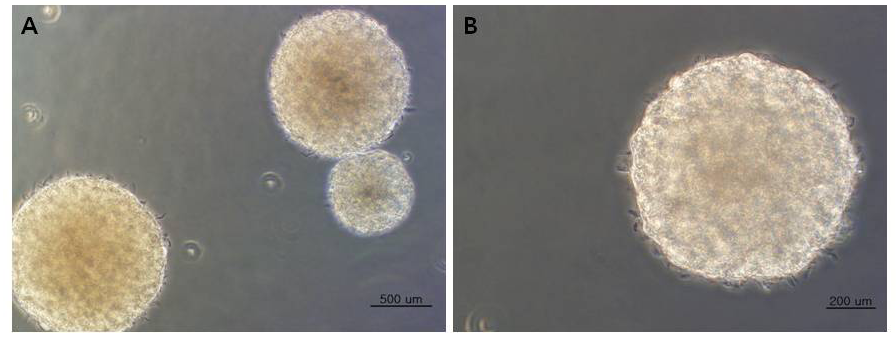

Supplement: Supplementary file 1 — 10.1186/s12935-016-0319-0 Culture of tumor spheres from processed specimens. [file 12935_2016_319_MOESM1_ESM.tif]
