# Supplementary material for: Failure of a patient-derived xenograft for brain tumor model prepared by implantation of tissue fragments
Source: Cancer Cell Int. 2016 Jun 10;16:43. doi: 10.1186/s12935-016-0319-0 (PMC4901492; doi:10.1186/s12935-016-0319-0)
Supplement: Supplementary file 2 — 10.1186/s12935-016-0319-0 Method for culture of tumor spheres from processed specimens. [file 12935_2016_319_MOESM2_ESM.docx]

**Supplementary Method**

**Sphere isolation from tumor specimen**

Fresh tumor specimens were obtained in the operating room from brain tumor patients undergoing surgery. Each specimen was place in a sterile centrifuge tube (SPL Life Sciences Co., Ltd., Korea) in ice, and weighed on the same electronic precision balance (Sartorius® TE4101-L, Sartorius Weighing Technology GmbH, Goettingen, Germany) within 1 hour. Thereafter, specimens were processed using the previously reported mechanical dissociation method [1-3]. Briefly, the surgical specimens were minced and dissociated with a scalpel in Dulbecco’s modified Eagle’s medium/nutrient mixture F-12 (DMEM/F-12; Mediatech, Manassas, VA, USA) and then passed through a series of 100-μm nylon mesh cell strainers (BD Falcon, Franklin Lakes, NJ, USA). The cell suspensions were then washed twice in DMEM/F-12 and cultured in complete medium (DMEM/F-12) containing 1xB27 supplements (Invitrogen, San Diego, CA, USA), 20 ng/ml of basic fibroblast growth factor (bFGF; Sigma, St. Louis, MO, USA), 20 ng/ml of epidermal growth factor (EGF; Sigma), and 50 U/ml penicillin/50 mg/ml streptomycin [1-3]*.*

**References**

1. Kong BH, Park NR, Shim JK, Kim BK, Shin HJ, Lee JH, et al. Isolation of glioma cancer stem cells in relation to histological grades in glioma specimens. Childs Nerv Syst 2013;29:217-29.

2. Shin GY, Shim JK, Lee JH, Shin HJ, Lee SJ, Huh YM, et al. Changes in the biological characteristics of glioma cancer stem cells after serial in vivo subtransplantation. Childs Nerv Syst 2013;29:55-64.

3. Sulman E, Aldape K, Colman H. Brain tumor stem cells. Curr Probl Cancer 2008;32:124-42.
